# Functional Traits of Male and Female Leaves of *Hippophae tibetana* on the Eastern Edge of the Tibetan Plateau and Their Altitudinal Variability

**DOI:** 10.3390/plants11192484

**Published:** 2022-09-22

**Authors:** Baoli Fan, Zongqi Ma, Pengfei Gao, Jing Lu, Nana Ding, Kun Sun

**Affiliations:** College of Life Science, North Western Normal University, Lanzhou 730070, China

**Keywords:** Tibetan plateau, altitude, dioecious, *Hippophae tibetana* Schlecht, leaf functional traits

## Abstract

To date, there have been few studies of the functional traits of the dioecious *Hippophae tibetana* Schlecht leaves, either male or female, in response to ecological factors such as altitude. Elucidating these relationships will establish an important scientific basis for vegetation restoration and reconstruction of the Tibetan Plateau ecosystem. The natural populations of *H. tibetana*, distributed across three field sites, at 2868 m, 3012 m and 3244 m, in Tianzhu, Gansu, were studied by field survey sampling and laboratory analysis. In particular, the adaptions of leaf functional traits to elevation in these dioecious plants were analyzed. The results show that: (1) there is no “midday depression” of photosynthetic activity in either male or female plants. Over a one-day period, the net photosynthetic rate (Pn) and transpiration rate (Tr) of *H. tibetana* female plants were higher than those of male plants (*p* < 0.05). This correlated to the period of vigorous fruit growth in the female plant. The measured Pn and Tr were maximal at the intermediate altitude (3012 m). The light compensation point (LCP) of the leaves of male and female plants were 57.6 and 43.2 μmol·m^−2^·s^−1^, respectively, and the light saturation points (LSP) of the leaves were 1857.6 and 1596.8 μmol·m^−2^·s^−1^. (2) Altitude had a significant effect on plant and leaf functional traits of male and female *H. tibetana* (*p* < 0.05), and no significant difference was noted between plants at the same altitude. The values for leaf area (LA), specific leaf weight (LMA), leaf phosphorus content per unit mass (Pmass) and leaf phosphorus content per unit area (Parea) were also maximal at the intermediate altitude. Leaf nitrogen content per unit area (Narea) and leaf nitrogen content per unit mass (Nmass) increased with altitude. This indicated that the functional traits of male and female plants and leaves of *H. tibetana* showed a strong “trade-off relationship” with altitude. (3) Pearson correlation analysis showed that there were significant correlations among functional traits of *H. tibetana* leaves. Redundancy analysis (RDA) showed that soil water content (SWC), altitude (Alt) and soil organic carbon (SOC) had significant effects on the functional traits of *H. tibetana* leaves (*p* < 0.05).

## 1. Introduction

Dioecious plants are an important component of terrestrial ecosystems. They account for 6% of all flowering plant species and are distributed across 959 genera of angiosperms [1]. Numerous morphological, physiological, and ecological differences between males and females are observed in the responses of many species to environmental stress [2]. Therefore, studying the variation in sexual dimorphism of dioecious plants under different conditions can help us understand the adaptation strategies of dioecious plants to environmental changes.

Plant functional traits are the morphological, physiological and phenological characteristics developed by plants in adapting to their respective environments [3]. As the most important organ for photosynthesis, respiration and transpiration in plants, leaf traits can be important indicators of the adaptation strategies of plants to environmental changes [4]. Currently, most studies are based on soft functional physical traits such as leaf water content (LWC), leaf area (LA) and specific leaf weight (LMA) because of the ease of measurement. In contrast, hard physiological traits such as net photosynthetic rate (Pn), transpiration rate (Tr), and water use efficiency (WUE) are more difficult to observe directly, although they provide a more accurate response to changes in the external environment [5]. Since soft and hard traits are closely related, their combination can better explain the resource acquisition mechanisms in different habitats.

Altitude, a key topographic factor, can induce large differences in water and heat partitioning on a smaller spatial scale [6]. Therefore, it is a natural laboratory for studying how plant leaf traits respond to the environment. During long-term evolution, alpine plants have developed specific characteristics in leaf morphology and physiology as they have adapted to their elevated habitat [7]. Ecologists have long conducted experiments on trends in leaf functional traits along an altitudinal gradient and have made a number of important findings. LMA has been found to increase with altitude, as has the Narea [8], while the Nmass decreased [9]. On the South Island of New Zealand, measurements in 118 species of plants [10] found that the total leaf nitrogen content decreased while leaf thickness increased with increasing altitude.

*H. tibetana* is a small perennial shrub with dioecious and parthenogenetic clonal reproduction, often forming large thickets, and is a pioneer species in community succession with important ecological and economic value. It is distributed across the Tibetan Plateau and Himalayan region at altitudes of 2800–5200 m in plateau grasslands, river floodplains and riverbanks [11]. Molecular phylogenetic studies by Sun et al. [12] have shown that *H. tibetana* is the most basal taxon of the entire genus Buckthorn and is one of the earliest differentiated species. This suggests that this species has been forced to gradually evolve in conjunction with the uplift of the Tibetan Plateau, adapting to the low oxygen and cold environment [13]. Previous studies on *H. tibetana* have dealt with the way in which morphological traits such as flowers, leaves and fruits are adapted to the high-altitude habitat of the Tibetan Plateau. However, as dioecious plants, the different adaptation strategies of female and male plants to high-altitude environments in terms of leaf morphology and physiology have been little studied. Therefore, in this study, the male and female plants of *H. tibetana*, a pioneer plant on the eastern edge of the Tibetan Plateau, were selected from three different altitudes to cover their alpine habitat, and their hard and soft leaf traits were measured along with key soil properties. The results will provide a scientific basis for vegetation restoration and reconstruction in the Tibetan Plateau ecosystem.

## 2. Results

### 2.1. Physical and Chemical Properties of Soil

The soil water content (SWC) at low-, middle- and high-altitude study sites (Tianzhu Tibetan Autonomous County, Gansu) was 21.52 ± 0.30%, 14.98 ± 0.24%, and 27.07 ± 0.20%, respectively. This was in line with the soil organic carbon (SOC) and soil total nitrogen (STN) in each area (Table 1) where high altitude > low altitude > middle altitude. The differences were all significantly different (*p* < 0.05). Soil total phosphorus (STP) at mid-altitude was significantly higher compared with the other two sites (*p* < 0.05). The soil pH at the low and middle altitudes was significantly more alkaline than that at the high-altitude site (*p* < 0.05). There was no significant difference in soil bulk density (SBD) between the different sites (*p* > 0.05). Overall, the high-altitude SWC, SOC and STN were higher than the lower two altitudes, with a minima at the middle-altitude site (Table 1).

### 2.2. Functional Traits of Leaves of Hippophae tibetana

#### 2.2.1. Photosynthetic Physiological Characteristics of Male and Female Plants of *Hippophae tibetana*

**Figure 1:**
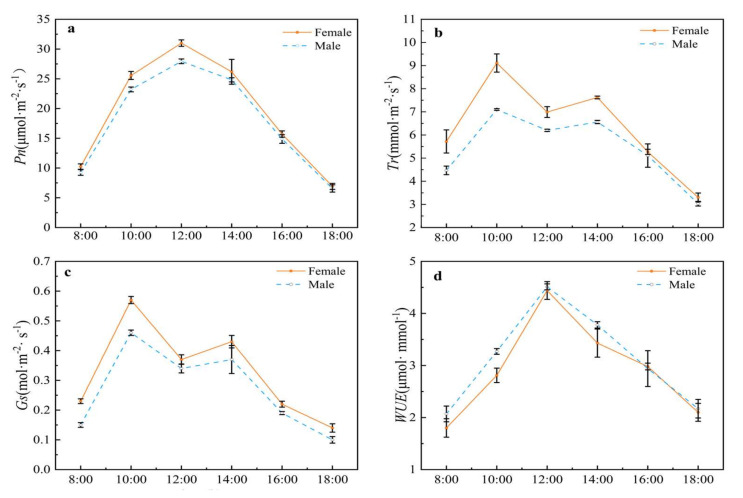

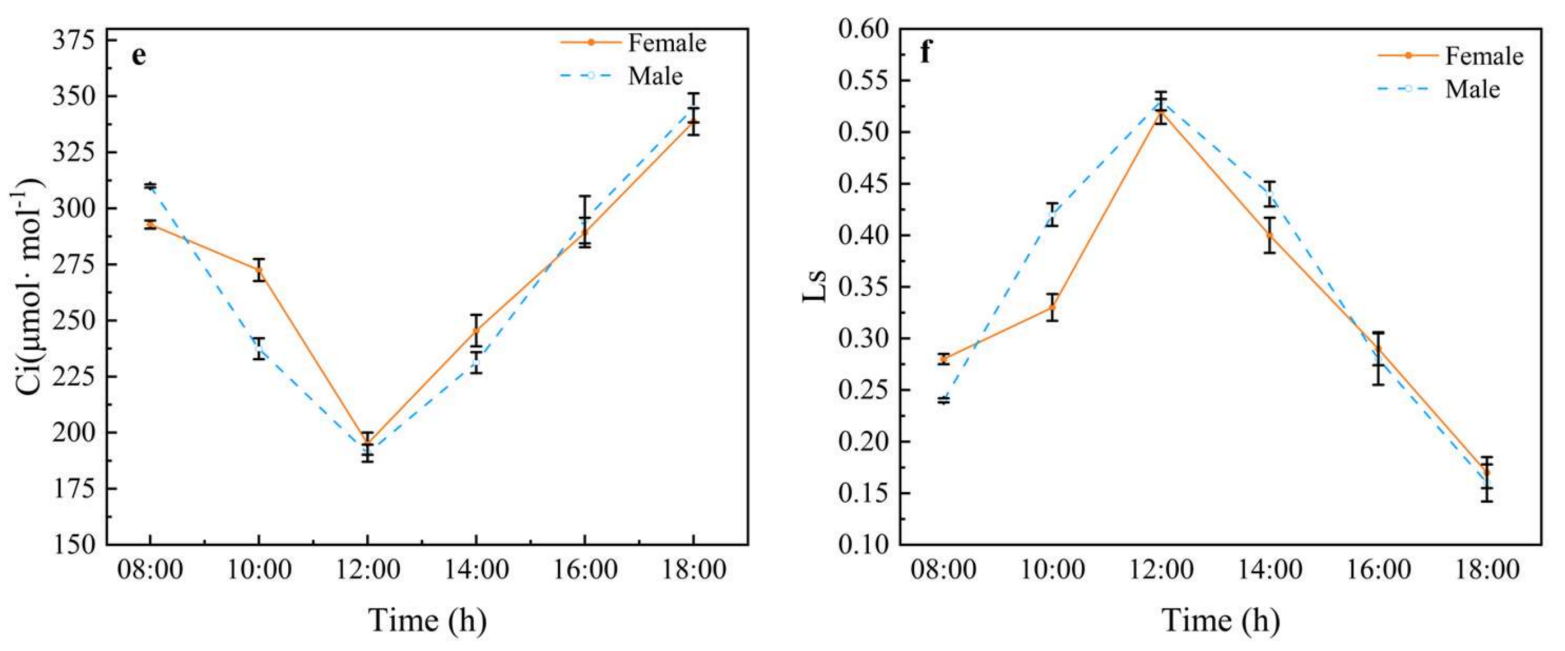
Daily variation of photosynthetic physiological indicators in male and female plants of *H. tibetana*. (**a**) Pn, net photosynthetic rate; (**b**) Tr, transpiration rate; (**c**) Gs, stomatal conductance; (**d**) WUE, water use efficiency; (**e**) Ci, intercellular CO_2_; (**f**) Ls, stomatal limitation value—all measured in early August 2021 (8.1–8.3) 8:00–18:00 h. The day length was about 14 h.

As shown in Figure 1a, a plot of the net photosynthetic maxima (Pn) for both male and female leaves of *Hippophae tibetana* showed a single maxima at midday, 31.00 and 27.94 μmol·m^−2^·s^−1^, respectively, rather than a bimodal peak corresponding to the phenomenon of the “photosynthetic lunch break”. Plots of both Tr (transpiration rate) (Figure 1b) and Gs (stomatal conductance) (Figure 1c) were essentially similar in form with both male and female plants achieving maxima at 10:00 h and 14:00 h. In both cases, the maxima for the female leaves were significantly higher than for the male leaves. The higher air temperature at midday and the stronger photosynthetic effective radiation caused the narrowing or closing of leaf stomata, resulting in the decrease in Gs, and the partial closure of the stomata, resulting in a reduced Tr (Figure 1b,c). The WUE of both male and female leaves of *H. tibetana* reached a maxima at midday (Figure 1d). Overall, the WUE of male plants was slightly higher than that of female plants throughout the day. As expected, the intercellular CO_2_ (Ci) (Figure 1e) declined to a minima at midday (190 mol.mol^−1^), corresponding to the stomatal closure (Ls 0.53, stomatal limitation value) (Figure 1f). The plots of both Ci and Ls were essentially identical for both male and female leaves. Some environmental information during the measurement is shown in Table 2.

#### 2.2.2. Photosynthesis-Light Response Curves of Male and Female Plants of *H. tibetana*

**Figure 2:**
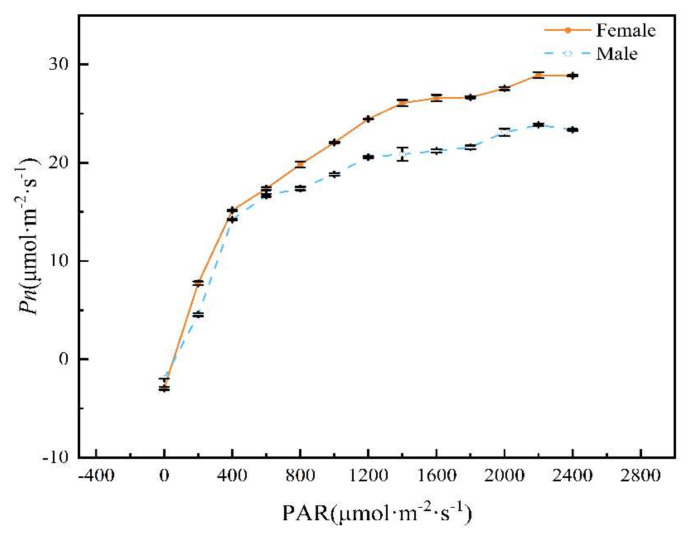
Pn-PAR curves of male and female plants of *H. tibetana,* all measured in early August 2021 (8.1–8.3) 8:00–18:00 h.

The Pn-PAR curves of male and female *H. tibetana* plants at middle altitude (Figure 2) demonstrate an initial rapid linear increase (A_max_) for both male and female plants from PAR 0 to 400 μmol·m^−2^·s^−1^. When the light intensity reached about 1800 μmol·m^−2^·s^−1^, the Pn of both male and female plants gradually saturated. This also corresponded with a concomitant increase in leaf temperature to a maxima at 14:00 h (Table 3).

The day length was about 14 h. The Pn-PAR parameters of male and female plants of *H. tibetana* were calculated using the light-response curve-fitting software Photosynthesis (Table 3, Figure 2). The photosynthetic parameters LSP and LCP are indicators of the ability of plants to use strong light and low light, respectively, and, in general, plants with higher LSP and lower LCP are more ecologically adapted to greater variations of light intensity [14]. In this study, the LCP of male and female plants of *H. tibetana* were determined to be 57.6 μmol·m^−2^·s^−1^ and 43.2 μmol·m^−2^·s^−1^, respectively. The LSP of female plants was found to be significantly higher than that of male plants (*p* < 0.05), with values of 1857.6 ± 61.28 μmol·m^−2^·s^−1^ and 1596.8 ± 44.60 μmol·m^−2^·s^−1^, respectively. Under certain environmental conditions, the maximum net photosynthetic rate (Amax) of leaves reflects the maximum photosynthetic capacity of plant leaves. The maximum daily changes in Amax and Pn were roughly similar in male and female plants, at 0.16 μmol·m^−2^·s^−1^ and 0.06 μmol·m^−2^·s^−1^, respectively. The apparent quantum efficiency (AQE), the initial slope of the photo-response curve (Figure 2), is an index reflecting the ability of plants to absorb, convert and utilize light energy under low light and is one of the indicators of light-energy conversion efficiency in photosynthesis. A high value indicates a high light-energy conversion efficiency of leaves [15]. The AQE values obtained by fitting the photosynthesis curve using the software Photosynthesis were all less than 0.125 (Table 3), indicating that the experimental results of the photo-response model were within the theoretical range. The AQE of female plants was significantly greater than that of male plants (*p* < 0.05), indicating that female plants have stronger light-energy conversion efficiency. The dark respiration rate (Rd) is the value of photosynthetically produced organic matter that is consumed by plants by respiration in the dark. Female plants had a higher dark respiration rate (*p* < 0.05), indicating that they need to consume more organic matter to maintain activity.

### 2.3. Altitudinal Changes in Physical Leaf Parameters of Male and Female H. tibetana Plants

#### 2.3.1. Functional Traits of Male and Female Leaves of *H. tibetana* at Different Altitudes

**Figure 3:**
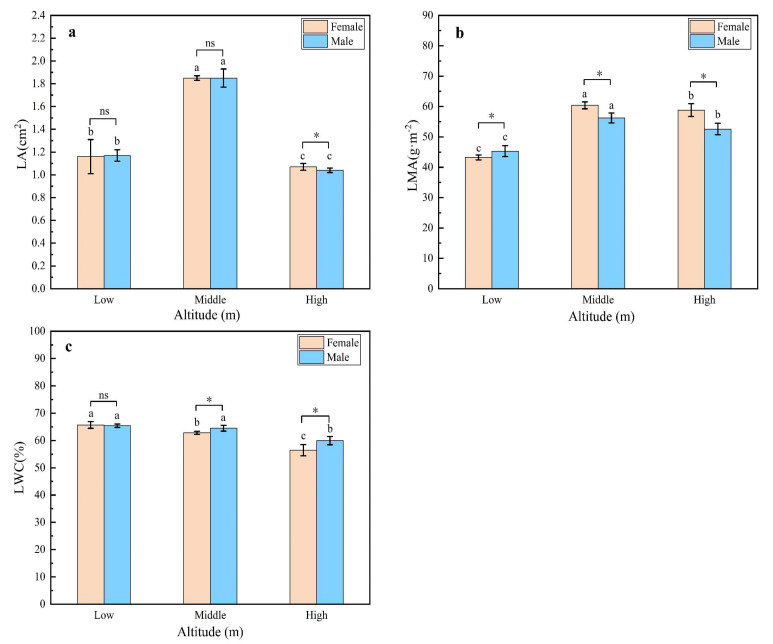
LA (leaf area), LMA (specific leaf weight) and LWC (leaf water content) of male and female leaves of *H. tibetana* measured at different altitudes. (**a**) LA of male and female *H. tibetana* at different altitudes. (**b**) LMA of male and female *H. tibetana* at different altitudes. (**c**) LWC of male and female *H. tibetana* at different altitudes. Different lowercase letters indicate significant differences (*p* < 0.05) between male and female *H. tibetana* at different elevation gradients, while “*” and “ns” indicate significant differences and no significant differences between male and female plants at the same elevation gradient, respectively.

The leaf area (LA), specific leaf weight (LMA) and leaf water content (LWC) of male and female plants were measured at three different altitudes (Figure 3). The LA was largest at the intermediate altitude with no significant difference (*p* < 0.05) between the male and female leaves (Figure 3a). This was also reflected in the LMA (Figure 3b), which was again shown to be greatest at the intermediate altitude but closely followed by the LMA at the highest altitude. Again, no significant differences between male and female leaves were observed. The LWC was found to be highest in the leaves from the lowest-altitude plants and no significant difference was observed between the male and female plants (Figure 3c). The LWC was found to decrease with altitude, most particularly for the female leaves, which had significantly lower LWC than the male leaves at the intermediate and highest altitudes.

**Figure 4:**
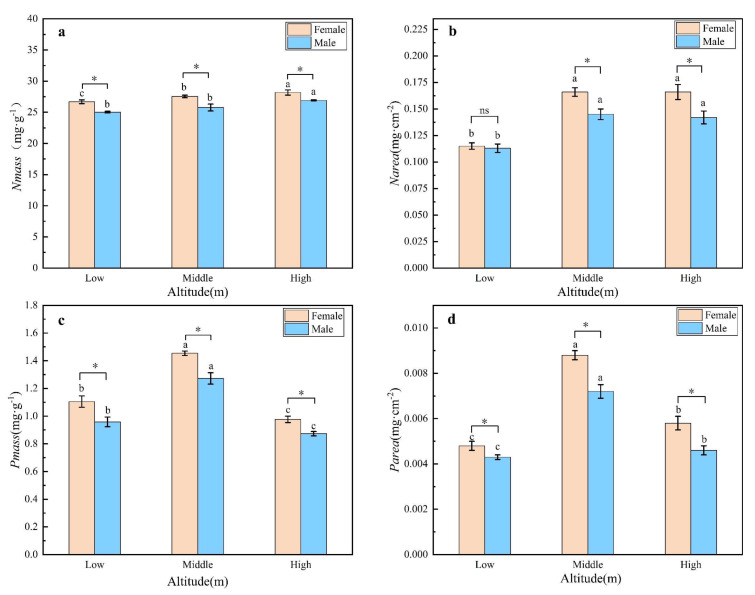
Nutrient indexes of male and female leaves of *H. tibetana* at different altitudes. (**a**) Nmass of male and female *H. tibetana* at different altitudes. (**b**) Narea of male and female *H. tibetana* at different altitudes. (**c**) Pmass of male and female *H. tibetana* at different altitudes. (**d**) Parea of male and female *H. tibetana* at different altitudes. **Nmass**, leaf nitrogen content per unit mass; **Narea**, leaf nitrogen content per unit area; **Pmass**, leaf phosphorus content per unit mass; **Parea**, leaf phosphorus content per unit area. “*” and “ns” indicate significant differences and no significant differences between male and female plants at the same elevation gradient, respectively. a, b, c indicate that there is a significant difference in the mean from large to small.

The nitrogen and phosphorus content of leaves was determined on a per mass (Nmass) and leaf area (Narea) basis. The Nmass of female plants was significantly higher than male plants across the three field sites and there was an overall trend of higher Nmass with increasing altitude (Figure 4a,b). These trends were more clearly seen when plotting Narea (Figure 4b). The phosphorus content of leaves was consistently higher for female plants compared to male plants at each of the field sites (Figure 4c,d). However, the Pmass (Figure 4c) and Pmass (Figure 4d) were maximal at the intermediate-altitude field site. 

In summary, the combined nutritional status, with respect to N and P, are optimal at the intermediate altitude with female plants tending to higher N and P content than male plants. 

#### 2.3.2. Photosynthetic Physiological Differences between Male and Female Plants of *H. tibetana* at Different Altitudes

**Figure 5:**
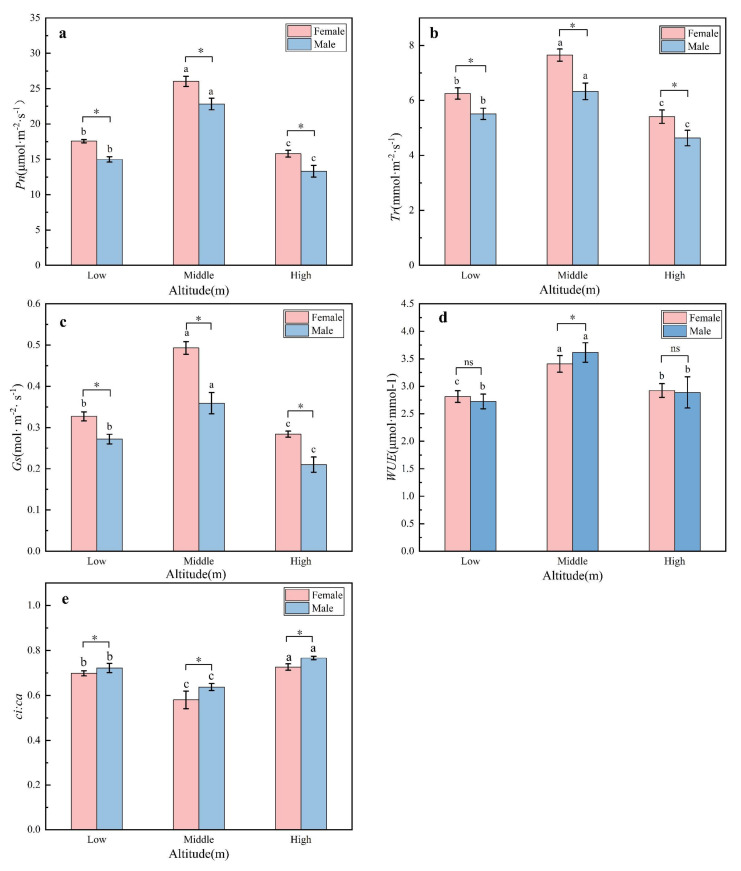
Photosynthetic parameters of male and female leaves of *H. tibetana* at different altitudes. (**a**) Pn at three altitude field sites; (**b**) Tr at three altitude field sites; (**c**) Gs at three altitude field sites; (**d**) WUE at three altitude field sites; (**e**) the ratio of intercellular CO_2_ concentration to atmospheric CO_2_ concentration (*ci:ca*) at three altitude field sites. To keep other environmental factors consistent, the three altitudes are set to the same photosynthetically active radiation PAR (1500 μmol·m^−2^·s^−1^). The leaf temperature is controlled at about 25 °C, the relative humidity was about 60% and the atmospheric CO_2_ concentration (about 400 μmol·mol^−1^) was adjusted with reference to the CO_2_ concentration. Other parameters are default values, and the time of measurement was between 9:00–11:30 a.m. “*” and “ns” indicate significant differences and no significant differences between male and female plants at the same elevation gradient, respectively. a, b, c indicate that there is a significant difference in the mean from large to small.

Under the same PAR (1500 μmol·m^−2^·s^−1^), the Pn (Figure 5a), Tr (Figure 5b), Gs (Figure 5c) and WUE (Figure 5d) of *H. tibetana* plants were maximized at the intermediate altitude with female plants achieving higher values than male plants. With respect to the *ci:ca* ratio (Figure 5e), this was at a minima at the intermediate altitude, indicative of an enhanced rate of photosynthesis. Again, female plants performed better than males across the three field sites.

#### 2.3.3. Correlation Analysis of Functional Traits of *H. tibetana* Leaves at Different Altitudes

**Figure 6:**
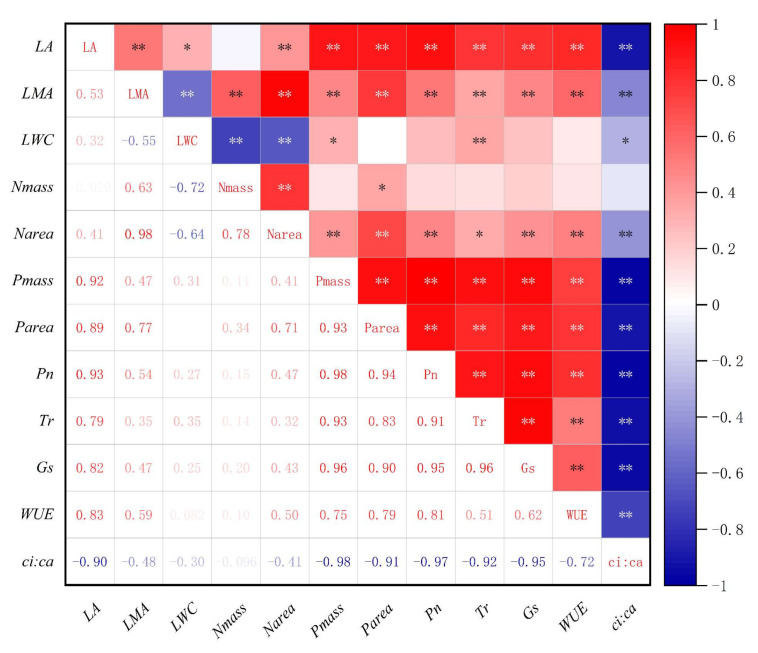
Correlation analysis of leaf functional traits of male and female plants of *H. tibetana* at different elevations. Note: ** indicates correlation is significant at *p* < 0.01 level; * indicates correlation is significant at *p* < 0.05 level.

**Figure 7:**
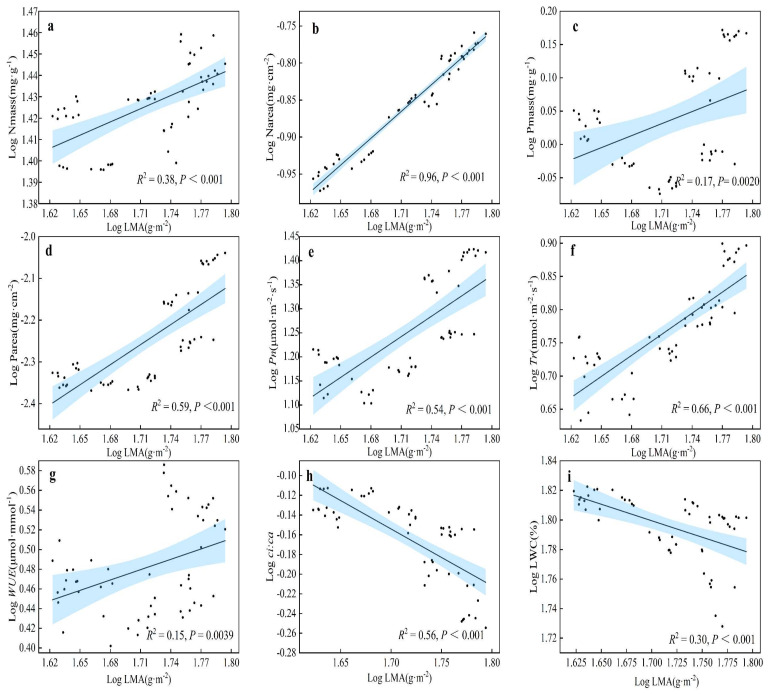
Linear regression relationship of LMA with other leaf functional traits. (**a**) Linear regression relationship between LMA and Nmass. (**b**) Linear regression relationship between LMA and Narea. (**c**) Linear regression relationship between LMA and Pmass. (**d**) Linear regression relationship between LMA and Parea. (**e**) Linear regression relationship between LMA and Pn. (**f**) Linear regression relationship between LMA and Tr. (**g**) Linear regression relationship between LMA and WUE. (**h**) Linear regression relationship between LMA and *ci:ca.* (**i**) Linear regression relationship between LMA and LWC.

**Figure 8:**
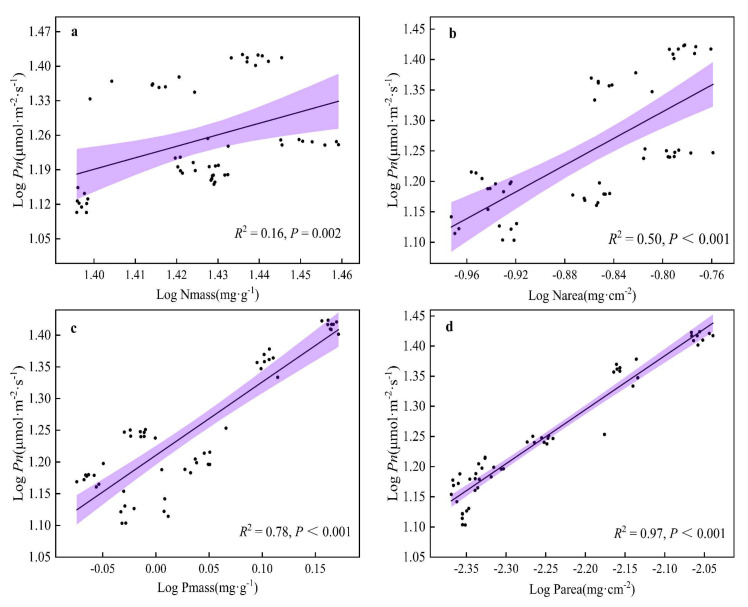
Linear regression relationship of Pn with other leaf functional traits. (**a**) Linear regression relationship between Nmass and Pn. (**b**) Linear regression relationship between Narea and Pn. (**c**) Linear regression relationship between Pmass and Pn. (**d**) Linear regression relationship between Parea and Pn.

**Figure 9:**
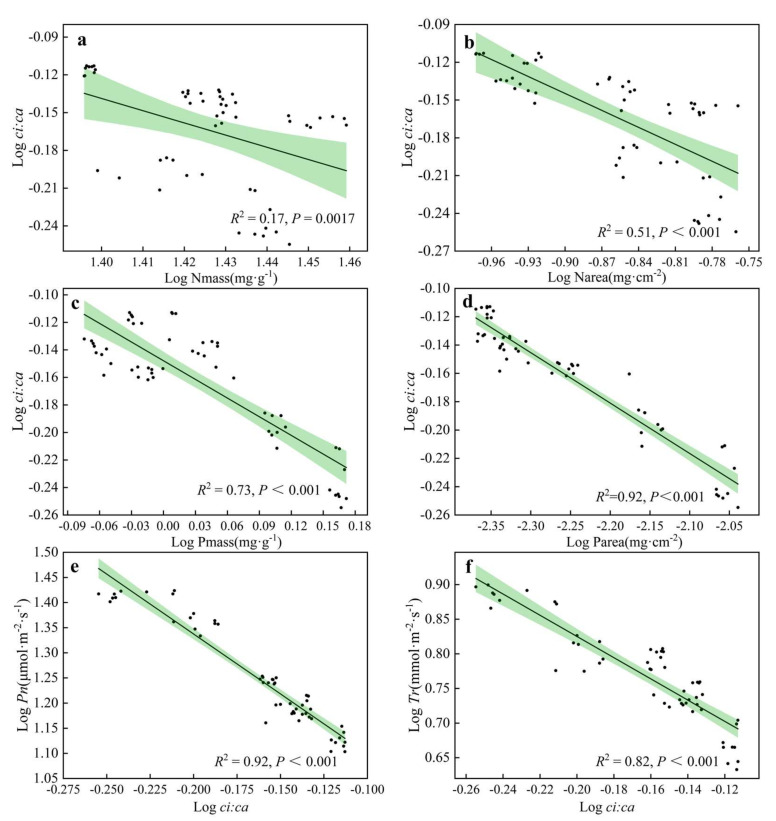
Linear regression relationship of *ci:ca* with other leaf functional traits. (**a**) Linear regression relationship between Nmass and *ci:ca*. (**b**) Linear regression relationship between Narea and *ci:ca*. (**c**) Linear regression relationship between Pmass and *ci:ca*. (**d**) Linear regression relationship between Parea and *ci:ca*. (**e**) Linear regression relationship between Pn and *ci:ca*. (**f**) Linear regression relationship between Tr and *ci:ca*.

A Pearson correlation analysis was performed on the functional traits of *H. tibetana* leaves (Figure 6), and indicators with significant correlations between leaf functional traits were selected for linear regression analysis (Figure 7, Figure 8 and Figure 9). LA was significantly positively correlated with LWC (*p* < 0.05), highly significantly positively correlated with LMA, Narea, Pmass, Parea, Pn, Tr, Gs and WUE (*p* < 0.01) and highly significantly negatively correlated with *ci:ca* (*p* < 0.01). LMA showed highly significant positive correlations (*p* < 0.01) with LA, Nmass, Narea, Pmass, Parea, Pn, Tr, Gs and WUE and highly significant negative correlations (*p* < 0.01) with LWC and *ci:ca* (Figure 6 and Figure 7). LWC showed significant positive correlation with LA and Pmass (*p* < 0.05), highly significant positive correlation with Tr (*p* < 0.01), significant negative correlation with *ci:ca* (*p* < 0.05) and highly significant negative correlation with LMA, Nmass and Narea (*p* < 0.01). Nmass showed a significant positive correlation with Parea (*p* < 0.05), a highly significant positive correlation with LMA and Narea (*p* < 0.01) and a highly significant negative correlation with LWC (*p* < 0.01). Narea was significantly positively correlated with Tr (*p* < 0.05), highly significantly positively correlated with LA, LMA, Nmass, Pmass, Parea, Pn, Gs and WUE (*p* < 0.01) and highly significantly negatively correlated with LWC and *ci:ca* (*p* < 0.01). Pmass was significantly positively correlated with LWC (*p* < 0.05), highly significantly positively correlated with LA, LMA, Nmass, Narea, Parea, Pn, Tr, Gs and WUE (*p* < 0.01) and highly significantly negatively correlated with *ci:ca* (*p* < 0.01). Parea was significantly positively correlated with Nmass (*p* < 0.05), highly significantly positively correlated with LA, LMA, Narea, Pmass, Pn, Tr, Gs and WUE (*p* < 0.01) and highly significantly negatively correlated with *ci:ca* (*p* < 0.01). Pn showed highly significant positive correlations with LA, LMA, Narea, Pmass, Tr, Gs and WUE (*p* < 0.01) and highly significant negative correlations with *ci:ca* (*p* < 0.01) (Figure 6 and Figure 8). Tr showed significant positive correlations with Narea (*p* < 0.05), highly significant positive correlations with LA, LMA, LWC, Pmass, Parea, Pn, Gs and WUE (*p* < 0.01) and highly significant negative correlations (*p* < 0.01) with *ci:ca*. *ci:ca* showed a significant negative correlation with LWC (*p* < 0.05), and a highly significant negative correlation with LA, LMA, Narea, Pmass, Parea, Pn, Tr, Gs and WUE (*p* < 0.01) (Figure 6 and Figure 7). In summary, leaf morphological traits, leaf nutrient elements and leaf photosynthetic physiological parameters were significantly correlated, indicating that leaf morphological traits and leaf nutrient elements play a key role in leaf photosynthetic capacity.

### 2.4. Redundancy Analysis between Leaf Functional Traits and Environmental Factors of H. tibetana

**Figure 10:**
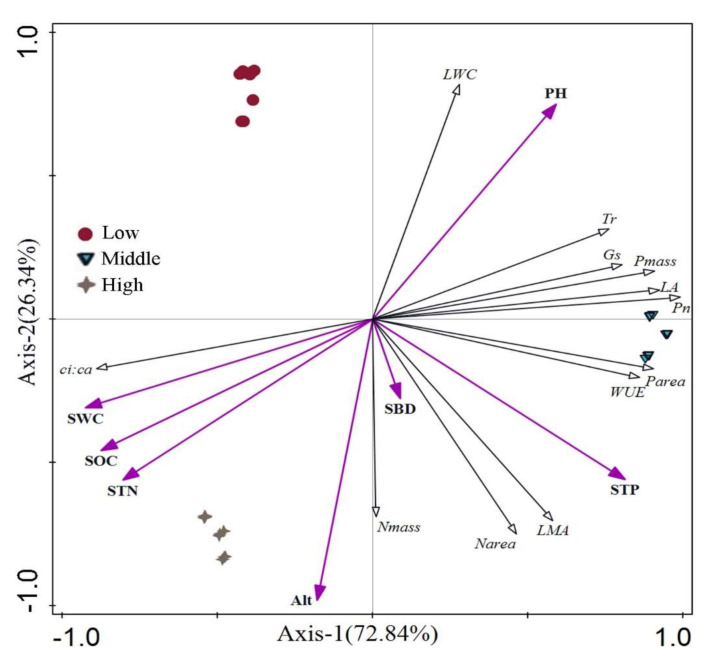
RDA ranking between functional traits of *H. tibetana* leaves and environmental factors. **Low**, low altitude. **Middle**, middle altitude. **High**, high altitude.

The correlation between environmental factors and functional traits of *H. tibetana* leaves was explored by performing redundancy analysis (RDA) to rank the functional traits of *H. tibetana* leaves against environmental factors (Figure 10). The eigenvalues of the first and second ranking axes were 0.5725 and 0.2070, respectively, and the two ranking axes together explained 77.95% of the variation in leaf functional traits and 99.18% of the leaf–environmental factor relationships, indicating that the first and second ranking axes could better reflect the relationships between leaf functional traits and environmental factors (Table 4). The correlation coefficients between leaf functional traits and environmental factors were 0.9427 and 0.9203 in the first and second ranking axes, respectively, indicating that leaf functional traits were closely related to environmental factors. A total of three significantly correlated environmental factors, SWC, altitude and SOC, were screened (*p* < 0.05) with a joint explanation of 77.4%, indicating that SWC, altitude and SOC are the main environmental factors affecting the functional traits of *H. tibetana* leaves. SWC, SOC and STN were positively correlated with *ci:ca* and Nmass, less correlated with Narea and LMA and negatively correlated with other leaf functional traits. Altitude was positively correlated with *ci:ca*, Nmass, Narea and LMA, less correlated with Parea and WUE and negatively correlated with other leaf functional traits. pH was positively correlated with LWC, Tr, Gs, Pn, Pmass, LA, Parea and WUE, less correlated with Narea and LMA and negatively correlated with other leaf functional traits. STP was positively correlated with LMA, Narea, Nmass, WUE, Parea, LA, Pn, Pmass, Gs and Tr, less correlated with LWC and negatively correlated with *ci:ca*. SBD was positively correlated with LMA, Narea, Nmass, WUE and Parea and less correlated with *ci:ca*, LA, Pn, Pmass and Gs and negatively correlated with LWC and Tr.

## 3. Discussion

### 3.1. Variation of Soil Physicochemical Factors among Altitudes 

Changes in altitude, an important topographic factor, causes changes in environmental factors such as climate, soil and vegetation community composition [16]. Although there have been many studies on the relationship between elevation and soil physicochemistry, the conclusions obtained often differ markedly. The soil pH is closely related to residue decomposition and precipitation [17]. In this study, we found that the soil pH became more acidic with increasing altitude, consistent with the results of Ma et al. [18]. Due to the increase in plant intermediate product components of alpine meadow composition vegetation at increasing altitude, which intensifies the acidic leaching process and gradually decreases the pH, SWC was lowest at the intermediate altitude, which is related to the topographic environment in which intermediate altitude meadows are located, which has a certain slope and is far from rivers, which is not conducive to water retention and water holding in meadows. Higher altitudes have higher SWC, probably because of lower temperatures and increased air humidity, resulting in lower soil evaporation and higher water content.

SOC is a direct manifestation of soil fertility, and its content and distribution characteristics have a significant impact on the distribution of regional STN and STP [19]. STN and STP are the most important nutrients for vegetation growth and development, as well as reproduction. It is generally believed that soil N is mainly derived from the decomposition of soil organic matter and has a consistent pattern of variation in spatial distribution at different altitudes. Numerous studies have shown that SOC and STN tend to increase with increasing altitude [20]. In this study, both SOC and STN were lowest at the intermediate-altitude field site, which is related to the regional geological characteristics, where SWC is lower, ground grass biomass is lower and biological residues and humus are less. It is generally believed that the soil’s total P content is usually controlled by the soil’s parent material and soil formation, so the total P content is slow to migrate in the soil and the spatial distribution is more stable. In this study, STP showed a pattern of increasing and then decreasing, with the highest total phosphorus in the intermediate-altitude soils, which was mainly caused by the geographical differences in soil material.

### 3.2. Photosynthetic Physiological Characteristics of Male and Female of H. tibetana

Photosynthesis is central to the growth and development of plants, and the strength of a plant’s photosynthetic capacity reflects its ability to adapt to its environment [21]. The results of this study showed that the daily trends of photosynthetic physiological indicators (Pn, Tr, Gs, WUE, Ci and Ls) were similar in both male and female plants of *H. tibetana*, and Pn, Tr and Gs were higher in female plants than in male plants throughout the day. Hultine et al. [22] suggested that the female’s characteristic of maximizing access to resources through photosynthesis is one way to compensate for her higher investment in reproduction. Obeso et al. [23] suggested that the fruiting period of some dioecious plants is associated with a relatively high photosynthetic rate of nearby leaves. In contrast, Wu et al. [24] showed that there was no significant difference in photosynthesis between male and female plants of *H. rhamnoides.* In this study, we found that both male and female *H. tibetana* plants showed a “single-peak” at the intermediate-altitude field site (3012 m), with the peak at 12:00 h. There was no obvious photosynthetic “lunch break” phenomenon in either male or female plants. This is mainly because the temperature at high altitude is lower at noon. Even if the light is stronger, it does not cause the photosynthetic “lunch break” of the leaves, and this photosynthetic characteristic of high-altitude plants may be related to the high activity of oxidative enzymes in the leaves. Farquhar et al. [25] concluded that there are two factors affecting Pn decline, stomatal restriction and non-stomatal restriction. The direction of change of stomatal restriction values Ls and ci is generally used as the basis and criterion for discrimination, where ci is the key indicator and when Pn and Gs decline; if Ls increases and ci decreases, it is stomatal restriction, and if Ls decreases and ci increases or remains unchanged, it is non-stomatal restriction. The results of this study showed that Ls decreased and ci increased when Pn decreased in *H. tibetana* at 12:00 h, indicating that the decrease in Pn at this time was mainly limited by non-stomatal factors.

The light response curve of a plant reflects the photochemical efficiency of the plant. Differences in photosynthetic parameters can reflect differences in the way different plants adapt to their environment and adaptability [26]. In general, plants with higher nutrient content have higher photosynthetic capacity and respiratory consumption and adapt to their environment through rapid nutrient cycling. This study has shown that Amax, LSP, LCP, AQE and Rd of female plants were significantly enhanced compared to male plants (*p* < 0.05). This may be related to the fact that female plants are in the fruit-forming stage and need more photosynthetic products for sexual reproduction. In general, the range of LCP for typical sun plants is 50–100 μmol·m^−2^·s^−1^ and the range of LSP is 1500–2000 μmol·m^−2^·s^−1^ [27]. In this study, the LCP and LSP of female plants of *H. tibetana* were significantly lower than for male plants (Table 3), thus favoring their photosynthetic ability. For well-grown plants, AQE values generally range from 0.040 to 0.070 [28]. The present study showed that the AQE of both male and female plants were located in this interval range, indicating that *H. tibetana* is highly adaptable to a high-altitude environment.

Changes in photosynthetic properties of alpine plants can reflect the adaptation strategies of plants to environmental conditions at high altitudes [29]. Photosynthesis and transpiration are the main physiological processes in plants. Pn effectively reflects the plant’s ability to assimilate CO_2_, and the increase in elevation gradient causes changes in external environmental factors, which in turn cause changes in plant Pn. In the present study, Pn of both male and female plants of *H. tibetana* were maximal at the intermediate field site, representing a playoff between better access to soil N and P on the one hand versus more intense light to drive photosynthesis at higher altitudes. In addition, the harsher environment at high altitude will also reduce the Pn and Tr of both male and female plants of *H. tibetana*. Zhang et al. [30] found that WUE did not change significantly with elevation in *Quercus pannosa*. Vats et al. [31]. showed that high-altitude areas have lower WUE and higher Tr. In this study, we found that the WUE values of leaves of both male and female plants of *H. tibetana* was maximal at the intermediate altitude. Gs is an important channel for gas exchange between the leaf and the outside world, and its opening degree directly affects the size of Pn [26]. The present study showed that the Gs of male and female plants was again maximal at the intermediate altitude, which is consistent with the results of the Wu et al. [32] study on Chinese sea buckthorn. This may be due to the variation in stomatal density and size previously reported by Li et al. [33]. The photosynthetic capacity of both male and female plants of *H. tibetana* at the intermediate altitude was better than that of high and low altitude. *ci:ca* is an important plant physiological and ecological characteristic value and, in addition to varying among species, is also influenced by environmental factors such as light, moisture and nutrition, and thus changes in *ci:ca* can be used to understand differences in the response of male and female plants to different altitudes. In this study, *ci:ca* was optimal at the intermediate altitude. The RDA analysis indicated that the *ci:ca* ratio a had strong positive correlation with SWC, SOC and STN, suggesting soil nutrients and moisture at different altitudes were the main contributing factors affecting *ci:ca*. In general, the photosynthetic capacity of *H. tibetana* in the mid-altitude field site is better than the other two altitudes, which is the result of the trade-off of various environmental factors. 

### 3.3. Adaptation Strategies of Leaf Functional Traits to Altitude in Male and Female of H. tibetana

Differences in altitude usually lead to corresponding changes in environmental factors such as light, soil and precipitation, resulting in uneven distribution of water and heat, making plant growth conditions more complex and causing complex adaptations of leaf traits to environmental conditions [34]. Most studies show that LA becomes smaller with increasing altitude [35]. In the present study, the LA of *H. tibetana* male and female plants was maximal at the intermediate altitude. This is consistent with the Lebrija-Trejos et al. [36] study. On the one hand, it may be related to the growth habit of *H. tibetana*, which may be affected by high-temperature stress at low altitude and low-temperature stress at high altitude. Second, according to the Pearson correlation analysis, both Pmass and Parea had extremely significant positive correlations with LA (*p* < 0.01), indicating that LA was greatly affected by the phosphorus content in leaves. RDA analysis showed that STP had a strong (positive) effect with Pmass, Parea and LA; thus, higher LA, Pmass and Parea at the intermediate-altitude site were associated with higher STP. LMA is often closely associated with plant growth and survival responses; it can reflect the adaptation characteristics of plants to different habitats, making it an important indicator in plant ecology research [8]. The results of studies on the response of LMA to altitude varied, with some studies showing an increase in LMA with increasing altitude. In contrast, some studies found that plant LMA was maximal at a particular altitude. In this study, the LMA of male and female plants was optimal at the intermediate-altitude field site. Except for the low elevation, where the LMA of male plants was greater than that of female plants, the female plants were greater than the male plants at both middle and high elevations, indicating that the female plants had a more sensitive response to environmental changes.

Nitrogen and phosphorus in the leaves are indispensable components of various proteins and genetic material and play an important role in the growth and development of plants [37]. The increase in Narea with elevation is a common phenomenon, but the trend of Nmass response to elevation is not consistent [38]. The response trends of Nmass and Narea of leaves of both male and female plants of *H. tibetana* in this study was to increase with altitude. Previous studies have also shown that as altitude increases, atmospheric temperature decreases and radiation increases so that not only does the dry matter input per unit area of the leaves of alpine plants increase but also the area of the leaves tends to decrease, leading to a consequent increase in Narea [39]. While the Pmass and Parea were clearly maximal at the intermediate-altitude site, the Nmass and Narea maxima were not so clearly defined [37]. The high STP at the intermediate altitude and its availability to plants through root uptake from the soil promoted the leaf phosphorus content of *H. tibetana*. We showed that Nmass, Narea, Pmass and Parea were greater in female plants than in male plants at different altitudes, and because of environmental stress plants have to allocate their limited nitrogen resources to tissues or structures that assist in adapting to the stressful growth environment, Therefore, female plants are highly adapted to high altitude environments.

### 3.4. Trade-Offs in Functional Traits of Male and Female Leaves of H. tibetana at Different Altitudes and Their Correlation with Environmental Factors

The trade-off relationship is the most common link between plant functional traits, also known as “ecological strategy”; that is, plants form the optimal combination of traits after natural selection [40]. In this study, Narea and Parea increased with the increase in LMA. As the LMA of plants increases, the leaves thicken, resulting in an increase in dry matter per unit area, which in turn leads to an increase in N and P content per unit area. Unlike the results of Keenan et al. [41], the present study found that Nmass and Pmass increased with increasing LMA, which may be related to the different tree species studied. Nmass is a key limiting factor for leaf photosynthetic capacity, and Pmass also has a large effect on leaf photosynthetic capacity. This study showed that both Nmass and Pmass were significantly and positively correlated with Pn (*p* < 0.01), so the leaf N and P contents were closely related to the photosynthetic characteristics of plants.

Plants regulate different functional traits according to environmental conditions to form the best combination of traits to maximize the use of resources and thus maximize their survival [42]. According to the results of the RDA analysis, the functional traits of *H. tibetana* leaves at different altitudes were influenced by environmental factors such as SWC, altitude, SOC, STN and STP. Of these, SWC, altitude and SOC have the largest influence. SWC can affect the basic functions of plant growth, development, reproduction, distribution and other physiological functions such as photosynthesis, soil fertility, soil heat and microbial activity. In this study, SWC had the greatest effect on the functional traits of *H. tibetana* leaves. The significant effect of altitude on plant LA, LMA, Nmass, Pmass and plant photosynthesis has been confirmed. In general, the mechanism of the effect of altitude on plant functional traits is that as altitude increases, plant growth and development are gradually limited by temperature and moisture, and thus the adaptation of plants to the environment will change significantly. SOC is mainly derived from plant, animal and microbial residues, and processes such as the input of plant litter can lead to differences in SOC content, which in turn affects the variability of plant functional traits. Chen et al. [43] showed that SOC exhibited a large effect on functional traits of *Pinus tabulaeformis* leaves. In this study, SOC was found to be the main soil factor affecting leaf functional traits. Because the variation in leaf functional traits of plants from different seed locations is influenced by a combination of multiple geographic environmental factors. In this paper, only environmental factors such as soil nutrients and elevation were selected to study the functional traits of leaves, and environmental factors such as latitude, longitude, average annual precipitation, average annual temperature, slope direction, relative humidity and cumulus temperature at the locations of various source species were not addressed. Therefore, further research in this area is needed.

## 4. Materials and Methods

### 4.1. Study Area

In August 2021, sample plots were selected at three different altitudes in the Jinqiang River basin of Zhuaxixiulong Town, Tianzhu County, where the distribution of *H. tibetana* is relatively concentrated, at the Zhuaxixiulong Scenic Area (102°48’ E, 37°09’ N, 2868 m), Honggeda Village (102°40′ E, 37°14′ N, 3012 m) and Shangbaishui Village (102°33′ E, 37°14′ N, 3244 m). These are described as low, intermediate and high altitudes, respectively, spanning 102°30′–102°50′ E, 37°05′–37°15′ N. This area is cold and humid, with a continental plateau monsoon climate. The average annual precipitation is 410.5 mm, with summer precipitation accounting for 59% of the annual precipitation. The average annual potential evaporation is 1592 mm, which is 3.8 times the annual precipitation. The average annual temperature is −0.1 °C, the accumulated temperature ≥0 °C is 1380 °C, the total annual sunshine hours is 2600 h and there is no absolute frost-free period. Across the three field sites, *H. tibetana* is the dominant species. Other plant species include *Anaphalis lactea*, *Polygonum viviparum*, *Saussurea hieracioides*, *Potentilla fruticosa*, *Carex capillifolia*, *Salix sinica*, *Festuca ovina*, *Artemisia annua*, *Galium aparinevar* and *Potentilla discolor*. Some photos from the field site will be uploaded to Supplementary Materials and named: Figure S1.

### 4.2. Sample Plot Setting and Sampling

In each field site, five male and five female *H. tibetana* plants were randomly selected. A soil auger was used to take soil samples down to 20 cm near each of the selected plants. The soil samples were placed in numbered self-sealing bags after removing the surface detritus. Weighed soil samples (m_1_) were placed in aluminum boxes and dried in an oven at 105 ℃ to a constant mass (m_2_), and the soil water content (SWC, %) was calculated:

SWC = (m_1_ − m_2_)/m_2_ × 100%

The soil bulk weight (SBD, g·cm^−3^) was calculated as:

SBD (g·cm^−3^) = m/v

where “SBD” is the soil bulk density, g·cm^−3^; “m” is the mass of dry soil in the ring knife, g; and “v” is the volume of the ring knife, cm^3^.

Soil total nitrogen (STN, g·kg^−1^) was determined by sulfuric acid–perchloric acid digestion and indophenol blue spectrophotometry [44]. Total phosphorus (STP, g·kg^−1^) content was determined by sulfuric acid–perchloric acid digestion and the molybdenum–antimony anti-colorimetric method [45]. Soil organic carbon (SOC, g·kg^−1^) was determined by the potassium dichromate a sulfuric acid oxidation method [46], and soil pH was determined using a pH electrode.

### 4.3. Determination of Leaf Functional Traits

In the laboratory, the leaves of male and female plants of *H. tibetana* were weighed for leaf fresh weight (LFW) using an electronic balance with an accuracy of 0.0001 g. The leaves were scanned with a CanoScan LIDE300 scanner, and the leaf area (LA) was calculated using the ImageJ software. Then, the leaves were dried at 80 ℃ for 24 h to constant mass and weighed to determine the leaf dry weight (LDW), leaf water content (LWC, %) and specific leaf weight (LMA, g·m^−2^). The calculations were as follows:

LWC (%) = (LFW (g) − LDW (g))/LFW (g) × 100%

LMA (g·m^−2^) = LDW (g)/LA (m^2^)

The phosphorus and nitrogen leaf contents were determined using the methods described in Section 4.2 (above). The Narea and Parea were calculated as follows:

Narea (mg·cm^−2^) = Nmass × LMA

Parea (mg·cm^−2^) = Pmass × LMA

### 4.4. Photosynthetic Physiological Measurements

The photosynthetic parameters of male and female plants of *H. tibetana* were measured at the beginning of August 2021 on three consecutive cloudless sunny days using a GFS-3000 portable photosynthesis–fluorescence measurement system (WALZ, Effeltrich, Germany). Three male and female plants of *H. tibetana* (described in Section 4.2) were selected in each of the three field sites. One vigorous leaf was selected in each of the three directions in the middle of each plant, and triplicate measurements were made to determine an average value. The three altitudes were set to the same photosynthetically active radiation PAR (1500 μmol·m^−2^·s^−1^), and the measurement time was 9:00–11:30 h. Since the leaves of *H. tibetana* were too small to fill the leaf chamber of the photosynthesis system, the numbered leaves were picked off after the experiment. The leaf area was calculated with graph paper, and the photosynthetic parameters were recalculated. The diurnal variation of photosynthesis was determined over a sunny day using the GFS-3000 photosynthesis−fluorescence measurement system. The measurement time was from 8:00 to 18:00 h. Data for the net photosynthetic rate (Pn), transpiration rate (Tr), stomatal conductance (Gs), intercellular CO_2_ concentration (Ci), photosynthetically active radiation (PAR) and atmospheric CO_2_ concentration (ca) was collected every 2 h in triplicate and averaged. Water-use efficiency (WUE) and stomatal limit value (Ls) were calculated as follows:

WUE = Pn/Tr, Ls = 1 − *ci:ca*

The photosynthesis−light response of the leaves was measured using the GFS-3000 photosynthesis−fluorescence measurement system. To keep other environmental factors stable and suitable during the observation process, the leaf temperature was set to 25 °C, the relative humidity was about 60%, and the reference CO_2_ concentration was adjusted to the atmospheric CO_2_ concentration (about 400 μmol·mol^−1^). Other parameters were set to defaults. Photosynthetically active radiation (PAR) was provided by artificial red and blue light sources, and the gradient was set to 2200, 2000, 1800, 1600, 1400, 1200, 1000, 800, 600, 400, 200 and 0 μmol·(m^−2^·s^−1^) and data collected in triplicate. The light-response curve-fitting software Photosynthesis was used to simulate the A-Q curve, the light compensation point (LCP), light saturation point (LSP), apparent dark respiration rate (Rd), maximum net photosynthetic rate (Amax) and apparent quantum efficiency (AQE). After previous investigation and research, we only selected the mid altitude as a typical model to study the diurnal photosynthetic changes and photosynthesis−light response curves of male and female plants of *H. tibetana*.

### 4.5. Statistical Analyses

The data were organized using Excel 2021. The Pn-PAR parameters were fitted using photosynthesis software (LI-COR, USA. https://www.licor.com/, accessed on 30 August 2022). SPSS 26.0 was used to perform one-way analysis of variance (ANOVA) for all leaf functional traits, and the least significant difference (LSD) method was used to compare differences between data groups. Pearson correlation analysis was used to test the relationship between leaf functional traits. Subsequently, linear regression analysis was performed for trait pairs, and the data were logarithmically (log) transformed to ensure that the data conformed to a normal distribution. RDA was performed using Canoco 5.0 to analyze the effects of environmental factors on functional traits of *H. tibetana* leaves. The results of the above data analysis were plotted using Origin 2022, and the data in the graphs are means ± SD.

## 5. Conclusions

This study has demonstrated that there is no “midday depression” phenomenon in either male or female *H. tibetana*. In addition, the measured photosynthetic parameters of female plants were significantly enhanced over those of the male plants; however, this is probably related to the fact that the female plants were undergoing vigorous fruit growth. “Non-stomatal restriction” was the main reason for the decrease in Pn in male and female plants at 12:00 noon. According to the LSP and LCP parameters of male and female plants, the *H. tibetana* is a positive plant. The functional traits of male and female plants and leaves at the intermediate altitude were higher than those at low and high altitudes, indicating that the *H. tibetana* had the strongest adaptability to the mid-altitude environment. The leaf functional traits of *H. tibetana* can make up for the deficiencies of habitat through certain trait variation and trait combination, as well as the interaction with environmental factors, showing a strong “trade-off” adaptation strategy to better adapt to the high-altitude environment.

## Figures and Tables

**Table 1:** Characteristic values of soil environmental factors at different altitudes.

Item	Low Altitude	Middle Altitude	High Altitude
SWC (%)	21.52 ± 0.30 ^b^	14.98 ± 0.24 ^c^	27.07 ± 0.20 ^a^
pH	7.59 ± 0.04 ^a^	7.55 ± 0.03 ^a^	6.56 ± 0.05 ^b^
SBD (g·cm^−3^)	0.78 ± 0.003 ^a^	0.79 ± 0.004 ^a^	0.78 ± 0.004 ^a^
SOC (g·kg^−1^)	42.61 ± 0.46 ^b^	33.67 ± 0.54 ^c^	54.37 ± 0.90 ^a^
STN (g·kg^−1^)	4.59 ± 0.020 ^b^	3.65 ± 0.076 ^c^	6.18 ± 0.020 ^a^
STP (g·kg^−1^)	0.45 ± 0.003 ^c^	0.65 ± 0.004 ^a^	0.55 ± 0.012 ^b^

**SWC**, soil water content; **pH**, soil pH value; **SBD**, soil bulk density; **SOC**, soil organic carbon; **STN**, soil total nitrogen; **STP**, soil total phosphorus. Different lowercase letters (a, b, c indicate that there is a significant difference in the mean from large to small) in the same row indicated significant differences between different elevation gradients (*p* < 0.05).

**Table 2:** Environmental photosynthetically active radiation (PAR) (μmol·m^−2^·s^−1^) and leaf surface temperature (T _leaf_) of photosynthetic diurnal variation of male and female *H. tibetana*, all measured in early August 2021 (8.1–8.3) 8:00–18:00 h. The day length was about 14 h. (a, b indicate that there is a significant difference in the mean from large to small).

Time (h)	PAR (μmol·m^−2^·s^−1^)		T _leaf_ (°C)	
	Female	Male	Female	Male
8:00	255.75 ± 1.23 ^b^	299.13 ± 38.89 ^a^	9.67 ± 0.01 ^b^	10.23 ± 0.12 ^a^
10:00	1495.41 ± 26.49 ^a^	1471.35 ± 10.18 ^b^	13.2 ± 0.05 ^b^	14.18 ± 0.10 ^a^
12:00	1797.26 ± 162.03 ^a^	1704.48 ± 52.98 ^a^	24.26 ± 0.07 ^a^	23.51 ± 0.53 ^b^
14:00	1753.87 ± 493.28 ^a^	1938.45 ± 40.94 ^a^	27.86 ± 0.01 ^a^	26.35 ± 0.06 ^b^
16:00	1583.02 ± 56.67 ^a^	1472.77 ± 21.17 ^b^	22.39 ± 0.01 ^b^	24.8 ± 0.04 ^a^
18:00	216.33 ± 29.16 ^a^	161.24 ± 4.55 ^b^	18.79 ± 0.04 ^a^	17.66 ± 0.08 ^b^

**Table 3:** Characteristic parameters of Pn-PAR in male and female *H. tibetana*.

	LSP	LCP	AQE	Rd	Amax
**Female**	1857.6 ± 61.28 ^a^	57.6 ± 1.23 ^a^	0.051 ± 0.002 ^a^	−2.96 ± 0.150 ^a^	31.16 ± 0.25 ^a^
**Male**	1596.8 ± 44.60 ^b^	43.2 ± 1.47 ^b^	0.046 ± 0.001 ^b^	−1.97 ± 0.006 ^b^	28.00 ± 3.47 ^b^

**LSP**, light saturation point; **LCP**, light compensation point; **AQE**, apparent quantum yield; **Rd**, apparent dark respiration rate; **Amax**, maximum assimilation rate. Different lowercase letters indicate significant differences between male and female plants (*p* < 0.05).

**Table 4:** RDA ranking of leaf functional traits and soil factors of *H. tibetana*.

Axis	Eigenvalues	Leaf–Environment Correlation Coefficient	Leaf Cumulative Percentage Variance (%)	Leaf–Environment Cumulative Percentage Variance (%)	Sum of All Eigenvalues	Sum of All Canonical Eigenvalues
Axis1	0.5725	0.9427	57.25	72.84	1.0000	0.7860
Axis2	0.2070	0.9203	77.95	99.18		
Axis3	0.0031	0.3068	78.27	99.58		
Axis4	0.0020	0.4124	78.46	99.83		

## Data Availability

Not applicable.

## Supplementary Materials

The following supporting information can be downloaded at: https://www.mdpi.com/article/10.3390/plants11192484/s1, Figure S1. Field altitude sites of *H. tibetana*. a, field habitats of *H. tibetana*. b, determination of photosynthesis of *H. tibetana* by photosynthesis. c, sample soil was collected. d, *H. tibetana* female. e, *H. tibetana* male.
