# Supplementary material for: Functional Traits of Male and Female Leaves of Hippophae tibetana on the Eastern Edge of the Tibetan Plateau and Their Altitudinal Variability
Source: Plants (Basel). 2022 Sep 22;11(19):2484. doi: 10.3390/plants11192484 (PMC9573225; doi:10.3390/plants11192484)
Supplement: Supplementary file 1 [file plants-11-02484-s001.zip › plants-1915967-supplementary.pdf]

Supplementary Data 1:

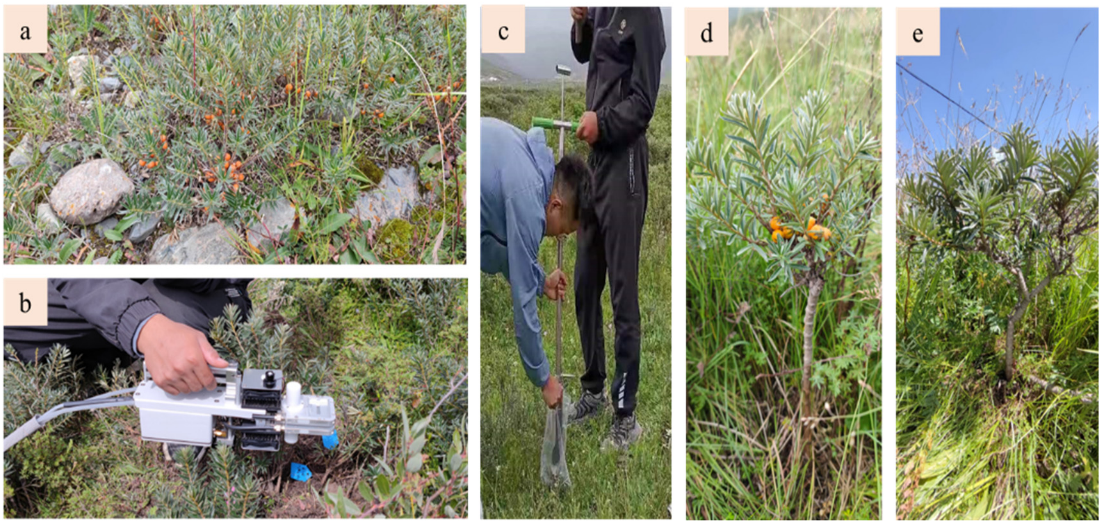

Figure S1. Field altitude sites of *H. tibetana*. **a,** field habitats of *H. tibetana.* **b**, determination of photosynthesis of *H. tibetana* by photosynthesis. **c**, sample soil was collected. **d**, *H. tibetana* female. **e,** *H. tibetana* male.
